# Practical Notes on Diagnosis and Treatment

**Published:** 1907-05-25

**Authors:** 


### PRACTICAL NOTES ON DIAGNOSIS AND TREATMENT.

**Insomnia.**

P. Pregowski recommends a warmed bed as a valuable sleep-producing agent. This recognition of its therapeutic virtues may, perhaps, lead to a revival of the old-fashioned warming-pan.

**Vulvitis in Infancy.**

A USEFUL lotion is warm milk and water, to each pint of which a teaspoonful of borax and a teaspoonful of liquor plumbi subacetatis have been added. This may be applied several times daily.—*Dr. Amand Routh.*

**Pigmentation of the Skin.**

VERY extensive pigmentation of the skin may occur in Graves' disease, and also sometimes in chronic Bright's disease. Hence this condition is not by itself sufficient for a diagnosis of Addison's disease.

**Salicylism and Epistaxis.**

DR. E. LEACH suggests that instances of epistaxis occurring in the course of influenza are due, not to the disease, but to the salicylate of sodium which is prescribed as a remedy. He quotes a case in which on three separate occasions the administration of salicylates was followed by epistaxis.

**Bone Disease in Syphilis.**

THE subjects of inherited syphilis are very prone to bone disease and often show severe forms of it; but it occurs in them with very considerable differences from those we observe in the acquired disease. In the latter it is often very late, say, after ten or twenty years of good health, and it does not usually affect many bones at the same time. In the subjects of inherited disease, on the contrary, it is almost invariably multiple, and often symmetrical, and it is met with in early youth and is transitory. *Mr. Jonathan Hutchinson.*

**Malignant Syphilis.**

IN severe cases of syphilis, and especially when the patient cannot readily take mercury or when the drug disagrees with him, sea-air should be prescribed. In itself it will not cure the disease, but it will enable the patient to take the remedy. Quinine and opium also have some value in the same direction.

**Sudden Death in Addison's Disease.**

SUDDEN death is one of the recognised dangers of Addison's disease. It is due to syncope and is particularly liable to occur in the more acute cases. Suddenly sitting up in bed may determine it.

**Treatment of Erysipelas.**

AS an internal remedy quinine in 4- to 8-grain doses, according to the temperature, may be given three or four times daily, and the following may be used as a local application:—Perchloride of mercury 1 grain, lanoline and vaseline, of each, half an ounce.

**Night Sweats.**

As the result of a series of observations with various drugs, Dr. Hy. Conklin came to the conclusion that agaricin is the most effective remedy in the treatment of the night sweats of phthisis pulmonalis. It can be given as a pill, dose $\frac{1}{6}$ to $\frac{1}{2}$ grain. A dose should be given late in the afternoon and a second dose four or five hours later.

**Constipation in Infants.**

COD-LIVER OIL is recommended by Dr. Brach, of Frankfort, as the best laxative in suckling children; with it a little sugar of milk may also be given. Gentle massage of the abdomen is another measure of great service in this condition. It should be applied three or four times daily, and for some five to ten minutes on each occasion.

**Temperature in Pleural Effusions.**

PYREXIA and hectic may be absent or little marked in empyema, especially in long-standing cases, for the alteration of the pleura or the degree of tension may prevent absorption. On the other hand, well-marked febrile temperatures may be present in serous effusions.

**Stimulants in Blood Poisoning.**

IN giving brandy you should give it not by tablespoonfuls but by teaspoonfuls, since it is better for a patient suffering from blood-poisoning that the necessary stimulant should be taken in small but repeated doses. I am quite sure of this, that I have seen cases which have been saved by stimulant treatment, particularly cases of scepticæmia, and occasionally a case of pyæmia.—*Mr. Christopher Heath.*

